# 40 Tesla miniature magnets

**DOI:** 10.1126/sciadv.adz5826

**Published:** 2026-03-11

**Authors:** Chukun Gao, Pin-Hui Chen, Nicholas Alaniva, James H. J. Ellison, Mairon Frei, Snædís Björgvinsdóttir, Edward P. Saliba, Yanhui Hu, Ioannis Pagonakis, Alexander Däpp, Ronny Gunzenhauser, Michael Urban, Klaus Ensslin, Alexander B. Barnes

**Affiliations:** ^1^ Department of Chemistry and Applied Biosciences, Eidgenössische Technische Hochschule Zürich, Zürich 8049, Switzerland.; ^2^ Resonance Exploration Technologies AG, Zürich 8044, Switzerland.; ^3^ Department of Physics, Eidgenössische Technische Hochschule Zürich, Zürich 8049, Switzerland.

## Abstract

Ultrahigh magnetic fields enable substantial advancements across a wide range of scientific disciplines. Traditionally, steady fields above 40 tesla have been achievable only with large resistive magnets that consume megawatts of power. Here, we demonstrate peak fields of 38 and 42 tesla using two compact all-HTS magnets, composed of two and four pancake coils, respectively. These magnets achieve current densities of up to 2257 amps per square millimeter and feature an exceptionally small bore diameter of 3.1 millimeters. Furthermore, the magnet coils are small enough to fit in the palm of a hand and consume less than 1 watt of power. Such compact magnets are realized through specialized winding methods that enable seamless winding over the small diameter while preserving the current-carrying capacity of the HTS tape. Nuclear magnetic resonance (NMR) experiments were performed inside the 3-millimeter magnet bore to provide calibration points for Hall sensors. These results show the potential of compact all-HTS magnets to enable widely accessible high-field NMR and other applications.

## Teaser

40 T magnetic fields are achieved within compact coils made of high-temperature superconductors.

## INTRODUCTION

High magnetic fields play an important role in enabling cutting-edge research and technology across a broad range of disciplines (*1*–*7*). For example, nuclear magnetic resonance (NMR) spectroscopy—one of the most powerful techniques for molecular structure determination—benefits from higher magnetic fields, which boost both sensitivity and resolution (*1*, *8*, *9*). This enhanced performance not only shortens measurement times but also enables the observation of previously unidentified molecular features that are undetectable at lower fields. Particularly for NMR studies of quadrupolar nuclei, NMR fields > 28 T are advantageous to improve spectral quality (*9*).

Magnetic fields exceeding 40 T can be achieved using high-temperature superconducting (HTS) tapes due to their exceptional tensile strength (*10*, *11*) and ability to maintain high in-field critical currents at cryogenic temperatures. In addition, the no-insulation (NI) winding technique (*12*–*17*), which omits turn-to-turn insulation, further enhances current density, enabling HTS coils to reach even higher magnetic fields. A notable example is the world-record 45.5 T steady-state magnet, which uses an NI HTS coil as an insert within a resistive background magnet (*18*–*20*). However, these high-field hybrid magnets are large and consume a substantial amount of power (>20 megawatt) (*21*, *22*).

In contrast, superconducting magnets consume far less power and can be more compact. Magnetic fields up to 32 T have been obtained using a hybrid approach with an HTS insert coil and a low-temperature superconducting (LTS) outer coil (*23*–*26*). Fully HTS-based magnets, without any background field, are advantageous for achieving fields beyond 40 T in a compact form. However, magnetic fields of up to 26 T have been demonstrated with all-HTS magnets, likely due to the high cost and engineering difficulties associated with wide bore (*27*–*30*).

We have been pursuing higher magnetic fields by developing diverse fabrication strategies and reducing the bore size (*31*–*33*). Here, we demonstrate high fields achieved in two compact HTS magnets wound with rare-earth barium copper oxide (REBCO)–coated conductor tape: one at 38 T composed of two pancake coils and one at 42 T composed of four (quad) pancake coils. The strong magnetic field achieved is attributed to the high current-carrying ability of REBCO and the extremely small magnet bore diameter of 3.1 mm. These magnets reached current densities of 2257 and 1880 Amm^−2^ at peak currents of 1246 and 1038 A, respectively. Despite the much higher current density, they consume a few thousand times less power and require a coil volume over 1000 times smaller than that of the 45.5 T hybrid magnet (*18*). We used a specialized winding technique to achieve a jointless connection between pancake coils at a winding diameter of 3.5 mm while preserving the integrity of the REBCO tape. To enhance the mechanical strength of the HTS magnet, we used a NI winding approach (*12*) combined with soldering. Last, we performed in situ NMR experiments at 4.2 K up to 20 T to calibrate the Hall sensor.

## RESULTS

### Construction of seamless double and quadruple (quad) HTS pancake coils

**Fig. 1.**
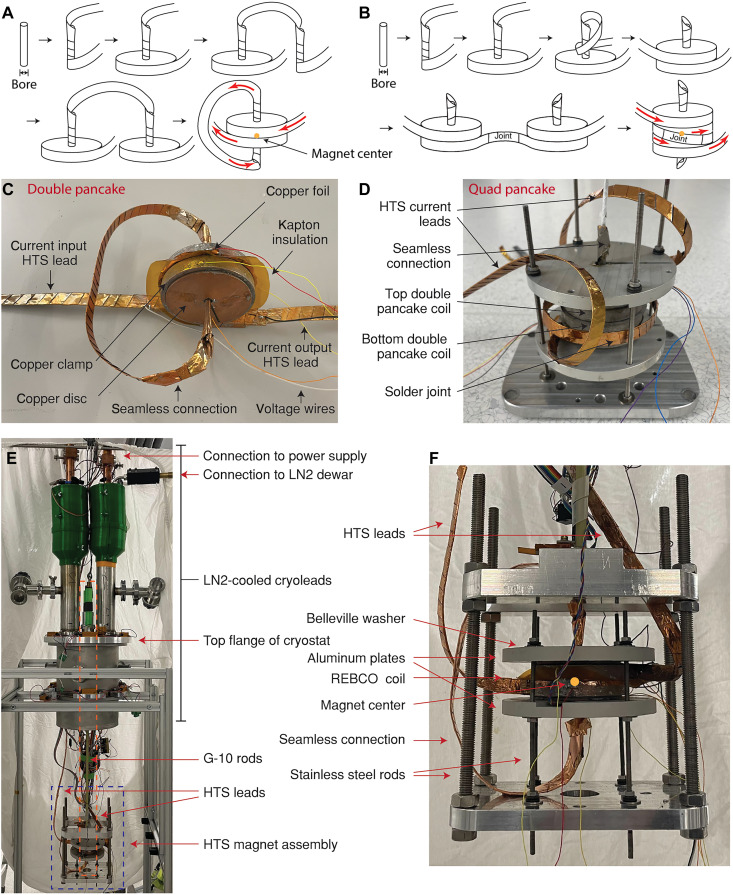
Winding, fabrication, and helium test setup of the seamless pancake coils. Schematic diagrams of the winding procedure for the seamless double (**A** and **B**) Quad pancake coil. Red arrows indicate the current flow. (**C**) Photo of the seamless double pancake coil after soldering. (**D**) Photo of the soldered seamless quad pancake coil installed in the helium test assembly. (**E**) Photograph of the helium test setup, showing the cryoleads (*33*) mounted on the top flange of the cryostat, with the magnet assembly suspended below the flange. The installation of the NMR probe setup is highlighted in the orange box, with a detailed view provided in Fig. 3A. (**F**) Expansion view of the magnet test assembly [highlighted in blue box in (E)], detailing its main components.

Coils wound using a continuous length of HTS tape avoid resistive joints in the current path, thereby improving coil performance and enabling higher current densities and magnetic fields. In a traditional seamless double pancake coil, in which the bore size is greater than the tape width, the seamless connection is realized through the innermost crossover turn between two coils (*34*, *35*). Other seamless winding strategies have also been reported (*36*, *37*). However, these methods work well only for narrow tapes (<6 mm) at relatively large winding diameter (>14 mm), so that the space required for the tilted inner turn is minimal. Here, the space at the 3.5-mm winding diameter is insufficient for the crossover turn of a 12-mm-wide tape (Table 1 and Fig. 1). Therefore, the seamless connection between the coils must be moved outside of the bore. We describe two winding methods to achieve an external seamless connection (Fig. 1, A and B). In the first method, the two coils are wound onto separate mandrels (Fig. 1A). The starting point for the winding is in the middle of the HTS tape. The innermost few turns are wrapped along the mandrel to transit the tape from a straight section into a coil form. The second coil is wrapped in the same way using the other half of the tape. Then, the seamless double pancake coil is formed by stacking the two coils together with the bottom side of each one facing the other (Fig. 1, A and C).

**Table 1.** Key parameters for the double and quad pancake coils.

Parameters	Values
**HTS tape**	
Manufacturer	SuperPower Inc.
Average critical current (77 K, self-field)	430 A
Length; width; thickness	137 and 269 m; 12 mm; 43 μm
Thickness of substrate/copper/*REBCO	30 μm/10 μm/1.5 μm
**Double pancake coil**	
Mandrel inner/outer diameter (mm)	3.1/3.5
Coil outer diameter (mm)	63.5/63.0
Height (mm)	28
Number of turns per coil	649/643
Max field and current	38.3 T at 1246 A
Current density (A/mm^2^)	2257
**Quad pancake coil**	
Mandrel inner/outer diameter (mm)	3.1/3.5
Coil outer diameter (mm)	62.8/62.8/62.6/62.8
Height (mm)	52
Number of turns per coil	628/643/635/629
Max field and current	42.3 T at 1038 A
Current density (A/mm^2^)	1880

* Rare earth elements: Gd/Y (50/50).

The coil is soldered using the same procedure as previously described (*31*, *32*) to enhance the mechanical strength and the current sharing ability between adjacent windings. The outermost few turns of each coil are released and used as HTS current leads connected to the liquid nitrogen–cooled copper charging leads (Fig. 1E) (*33*). Both the current leads and the seamless connection are sandwiched by additional HTS tapes and a 0.5-mm-thick copper strip to improve the mechanical strength, thermal conductivity, and tolerance to overcurrent. Copper foils are wrapped around the HTS leads to hold the stacked tapes and copper strip together. Solder paste is applied to the coil surface, and each pancake coil is encapsulated in pretinned copper surrounding parts, including a C-shape copper ring to hold the coil form, a 2-mm-thick copper disc on the top surface, and a copper foil on the bottom surface. A 50-μm-thick Kapton foil is sandwiched between the two coils for electric insulation. The double pancake coil assembly is baked at 170°C for 1 hour. Figure 1C shows the double pancake coil after soldering.

The quad pancake coil consists of two seamless double pancake coils, which are wound using the second method and then joined together (Fig. 1B). The first pancake and seamless turn are made the same as in method 1. However, instead of placing the second pancake beneath the first, the HTS tape is wrapped back over the seamless connection to form the second pancake above the first. This results in a configuration similar to the traditional seamless double pancake design but with a key difference: The crossover turn is replaced by an external seamless connection, achieved by wrapping the HTS tape around an extended mandrel. The other double pancake coil is constructed using the same approach and connected to the first via a solder joint. The two double pancakes are then stacked with the solder joint lying flat around the outer surface of the magnet. This joint is further reinforced with additional HTS tapes for enhanced mechanical and electrical stability (Fig. 1D). Although a fully seamless connection between the two double pancakes is feasible (*37*), it is not implemented here due to the limited conductor length. The final quad pancake assembly uses the same soldering method as that used in the double pancake coil.

### Liquid helium experiment setup

Figure 1 (E and F) depicts the setup for the helium test. The cryoleads (*33*) can transfer over 2 kA of current from the room temperature power supply to 77 K, cooled by continuous liquid nitrogen flow (Fig. 1E). The bottom of each cryolead is connected to a stacked HTS lead, which delivers current to the HTS magnet submerged in liquid helium at 4.2 K without causing significant helium boil-off. The HTS magnet assembly is held with two G-10 rods from the cryostat’s top flange. The HTS coil is secured with two aluminum plates and four M4 stainless steel rods (Fig. 1F). During the helium tests, voltages are measured using coated copper wires soldered onto the coil, and the temperature is recorded using Cernox sensors affixed to the coil. The Hall sensor was calibrated up to 28 T at 300 K in commercial NMR magnets and up to 33 T at 4.2 K using both NMR and the quantum Hall effect. The Hall response remained linear within uncertainty up to 42 T, consistent with previously reported NI REBCO coil data (*18*). Although an NMR-based field validation is not yet available, the combined calibration and linear response across the full operating range support the reliability of the field values reported here.

### Small coil validation

To explore the feasibility of the winding technique on extremely small bending diameters, two smaller double-pancake coils were fabricated and tested before the larger coils presented in this manuscript (figs. S1 and S2). These prototype coils, wound on 5- and 3.5-mm mandrels using 20 and 60 m of REBCO tape, respectively, served as an intermediate step between our previously reported mini magnets (*32*) and the present work using much longer conductors. They reached central fields of 23.9 T at 1656 A and 30.2 T at 1246 A, corresponding to current densities of 3000 and 2305 A/mm^2^, respectively. The resistance over the seamless connection at a bending diameter of 3.5 mm was measured in the coil configuration during liquid helium experiments. Resistance of less than 1 nΩ was measured at 1246 A and 30 T (fig. S2D), indicating that the critical current of the HTS tape under these conditions exceeds 1200 A. This observation is consistent with prior studies reporting a critical bending radius of less than 4 mm (*38*–*40*). The ability to reach relatively high fields while maintaining REBCO integrity with this winding approach motivated the fabrication of the larger double- and quad-pancake coils presented in the main study.

### Liquid helium test results of the double pancake coil

The following parameters were recorded throughout the charging experiments: power supply current (*I*_ps_), top and bottom pancake coil voltages (*V*_top_ and *V*_bottom_), coil temperature (*T*), and axial center magnetic field (*B*_z_). During the helium test, we paused charging for extended periods and fit *V*(*t*) to an exponential decay function to extract the decay constant, which serves as a metric for evaluating field stability and effective coil resistance.

**Fig. 2.**
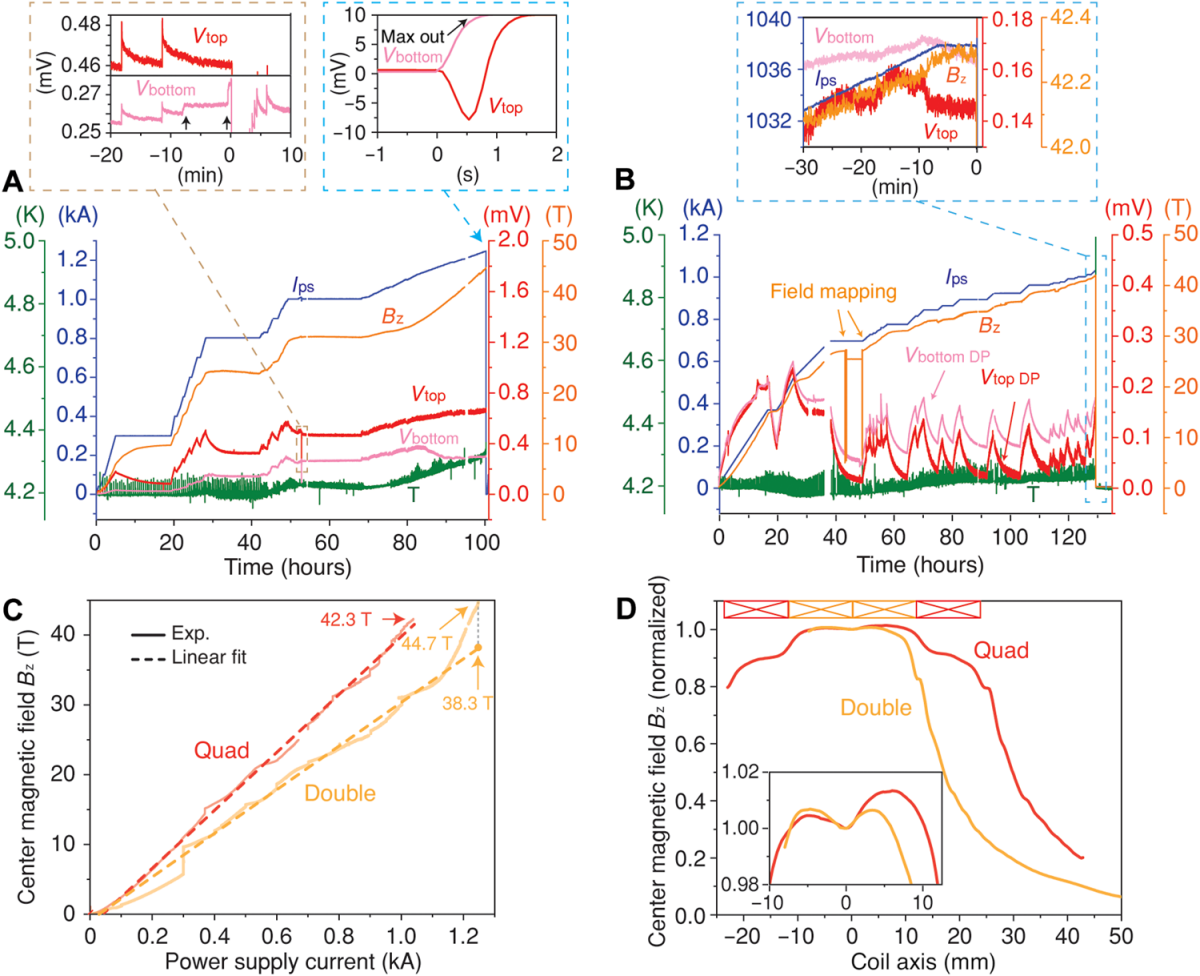
Liquid helium test results. The power supply current (*I*_ps_), coil voltages, coil temperature (*T*), and center magnetic field (*B*_z_) were measured during the tests for the (**A**) double and (**B**) quad pancake coils. For the double pancake coil, voltages across the top (*V*_top_) and bottom (*V*_bottom_) pancakes were recorded. For the quad pancake coil, the voltages across the top (*V*_top DP_) and bottom (*V*_bottom DP_) double pancake units were measured. The *x* axes of the inset plots are shown on finer time scales (in minutes or seconds), with the zero point shifted to align with the quench onset, to provide a clearer view of the transient behavior. Voltage above 10 mV, during quench, is outside the dynamic range of measurement. (**C**) Magnetic fields of both coils measured by the Hall sensor as a function of power supply current. Dashed lines represent linear fits to the data. (**D**) The magnetic field profiles of both coils were measured by moving the Hall sensor along the coil axis.

The test results for the double pancake coil in liquid helium are shown in Fig. 2A. The charging rate was 0.02 A/s at the beginning and then reduced gradually to 0.002 A/s above 1000 A until the magnet quenched. The current was kept constant for over 14 hours at 300, 800, and 1000 A for the voltages and magnetic field to stabilize. The relatively long charging and stabilization times are typical for NI coils due to the coil inductance and contact resistance between coil windings (*12*, *41*–*44*). The voltage decay time constant at 300 A was 5.26 hours, more than twice the value observed at 800 A (table S2). This could be attributed to a change in the contact resistance between coil windings that resulted in a longer charging delay at low currents (*12*, *44*, *45*). Superconducting magnets exhibit a voltage during charging due to coil inductance, which vanishes as the current reaches a steady value. The relatively high stabilized coil voltages observed in the double pancake coil suggest the presence of localized resistive regions, potentially caused by mechanical or thermal damage to the REBCO layer during coil fabrication (*28*). Some voltage instabilities were detected around 52 hours (expansion view in Fig. 2A), which were tentatively ascribed to local heating or movement within the coil. At this point, the current was reduced to avoid the propagation of a potential quench (*31*). The magnetic field drift measured at 1000 A was 350 parts per million (ppm)/hour (fig. S7A). We suggest that longer equilibration times, compensation coils, current sweep cycles, and temperature change cycles (*46*) could be used to reduce the drift rate.

The magnet charging was resumed at a much lower rate of 0.002 A/s after the overnight stabilization at 1 kA. After 80 hours, the magnetic field showed a much faster increase with current until the quench at 45 T, while the bottom coil showed a simultaneous voltage drop (Fig. 2A). When the magnetic field is plotted as a function of power supply current, it shows a linear increase in the region from 0.3 to 1 kA (Fig. 2C). The deviation below 0.3 kA was due to charging delay (table S2), while the faster increase after 1 kA is attributed to two reasons: (i) the current redistribution, which was indicated by the simultaneous voltage drop; and (ii) the quantum Hall effect, which can introduce nonlinearities in Hall sensor response under extremely low temperature and high field. As detailed in the Supplementary Materials (fig. S9), accurate Hall resistance plateaus were measured, confirming the magnetic field of 33 T at the highest quantized level. A magnetic field of 38 T at the quench current was extrapolated on the basis of linear fitting of these quantized levels. The faster rise above 1 kA observed in Fig. 2A could arise from a measurement deviation due to the quantum Hall effect, which occurred because of the thermal disturbance in the Hall sensor (*32*, *47*, *48*). The exact nature of these effects depends on the design and type of Hall sensor used. Further investigations are necessary to understand these influences. Therefore, we report a peak field of 38.3 T at 1246 A based on a constant field-per-current slope extrapolated from the linear regime before deviation.

During quench, a negative voltage was measured on the top coil before both coil voltages increased beyond the measurement limit (expansion view in Fig. 2A). This indicates that the quench was initiated in the bottom coil and propagated by inductive coupling to the top coil within seconds (*18*, *49*, *50*). The total energy stored in the compact double pancake magnet is ~18 kJ. In comparison, wide-bore magnets operating at lower fields store much higher energy due to the large inductance, for example, 8.6 MJ in the 32 T LTS-HTS magnet (*51*) and 370 kJ in the 26 T all-HTS magnet (*27*). During a quench event in our experiment, only ~12 liters of liquid helium was boiled off. In addition, the small-bore HTS magnets enable much higher magnetic fields while using substantially less HTS tape. For instance, the 26 T all-HTS magnet used 4.8 km of HTS tape (*27*), whereas only 137 m is needed for the 38 T magnet in this study (table S3).

### Liquid helium test results of the quad pancake coil

The power supply current (*I*_ps_), voltages of the top and bottom double pancake coil unit (*V*_top DP_ and *V*_bottom DP_), coil temperature (*T*), and axial center magnetic field (*B*_z_) of the quad pancake coil were recorded and plotted in Fig. 2B. The overall charging of the quad pancake coil was slower (0.006 to 0.0036 A/s) with more frequent pauses for the voltage and magnetic field to stabilize. This quad pancake coil reached a maximum magnetic field of 42.3 T after 125 hours of charging and then quenched during a pause at 1038 A (expansion view in Fig. 2B), which corresponds to a current density of 1880 A/mm^2^.

The slower charging rate used for the quad coil at the start of the experiment led to a good linearity between the magnetic field and the power supply current (Fig. 2C). No signs of the quantum Hall effect were observed before the quench. The absence of the quantum Hall effect in the quad coil was due to improved thermal insulation of Hall sensor, which minimized temperature fluctuation. The quad pancake coil showed 32% higher magnetic field than that of the double pancake coil at the same power supply current and quenched at a lower current, owing to the reduced critical current at higher fields.

The magnetic field profile was measured for both the double and quad pancake coils by translating the Hall sensor stepwise along the coil axis (Fig. 2D). Both profiles show a local minimum in the coil center and two local maxima nearby. Because of the additional two pancake coils, the profile for the quad coil shows two plateaus besides the local maxima. The distorted magnetic field profiles were mainly due to the screening current generated in REBCO tapes (*10*, *52*–*54*). The screening current–induced field in the magnet center is relatively strong in these mini magnets (fig. S3), due to the short distance between the screening current and the magnet center. The magnetic field homogeneity measured by the Hall sensor over a 0.6-mm region along the coil axis was ~100 to 180 ppm at the local minimum for both coils.

### NMR measurements and Hall sensor calibration

**Fig. 3.**
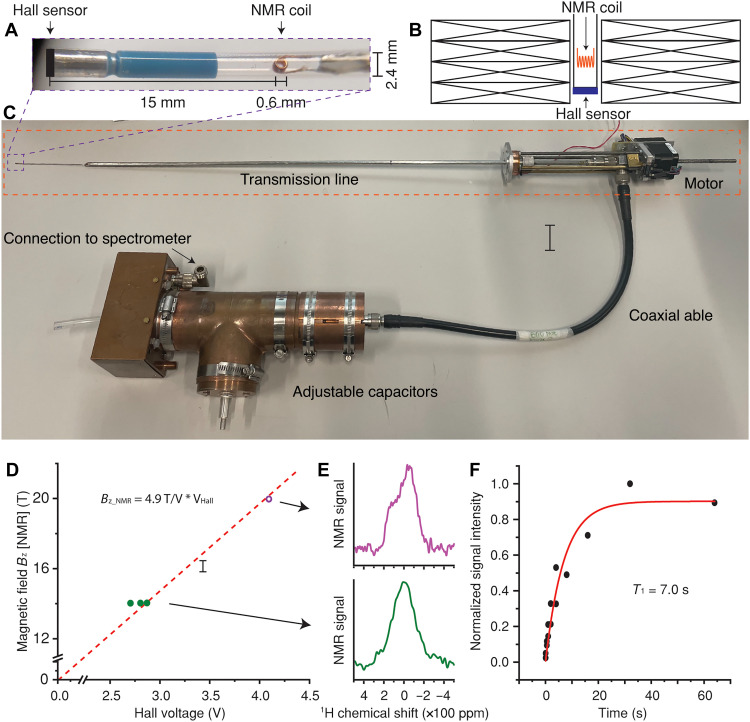
NMR measurements within the 3.1-mm cold bore of the double pancake coil in liquid helium. (**A**) Expansion view of the Hall sensor, 2.4-mm-diameter quartz sample tube, and a 0.6-mm-diameter five-turn solenoid NMR coil. (**B**) Schematic diagram illustrating the integration of the NMR coil and Hall sensor within the HTS magnet. (**C**) Customized transmission line probe. The main elements of the probe include adjustable capacitors, a coaxial cable, a copper transmission line, and an NMR coil. The transmission line was tapered at the terminal to fit within the small magnet bore. (**D**) Calibration points of the Hall sensor at 14 and 20 T. The dashed line indicates a linear fit with slope of 4.92 ± 0.04 T/V. (**E**) ^1^H NMR spectra measured at 597.463 MHz (14.0325 T, top) and 850.182 MHz (19.9681 T, bottom). (**F**) ^1^H T_1_ curve acquired at 14 T and 4.2 K.

NMR can precisely measure the magnetic field with an accuracy of a few parts per million and is commonly used as a reference for calibrating magnetometers (*55*, *56*). We performed NMR experiments within the 3.1-mm cold bore of the double pancake coil to calibrate the Hall sensor at 4.2 K (Fig. 3). A customized transmission line NMR probe (*57*) with an NMR coil and a Hall sensor attached 15 mm away was used for NMR experiments (Fig. 3A). The motor installed on top of the cryostat allows the adjustment of the vertical position of the probe, so the NMR coil and Hall sensor can be moved to the coil center for the NMR measurement and in situ calibration, respectively (Fig. 3B).

For the NMR measurements, the power supply current was slowly ramped, while NMR signal was constantly acquired at a fixed frequency. For example, to measure the ^1^H NMR spectrum at 597.463 MHz (corresponding to 14.0325 T), the HTS magnet was first charged to ~13 T. After the magnetic field stabilized for several hours, the NMR coil was moved to the magnet center, and the current was gradually increased while spectra were continuously recorded until the NMR signal appeared. After the acquisition, the Hall sensor was moved back to the magnet center for calibration.

^1^H NMR experiments using a single excitation pulse were performed on water glycerol ice sample [60% ^13^C-glycerol + 40% H_2_O) containing 40 mM 4-oxo-TEMPO (4-oxo-2,2′,6,6′-tetramethyl(piperidin-1-oxyl)]. The TEMPO radical was intended to reduce the relaxation time (T_1_) and thus shorten the signal acquisition time of ^1^H at 4.2 K (*58*, *59*). Spectra were recorded at 14 and 20 T using commercial 600- and 850-MHz spectrometers, respectively (Fig. 3, E and F). The full width at half maximum (FWHM) of the ^1^H NMR signal was measured to be ~270 ppm, containing both homogeneous and inhomogeneous components. However, because the signal is too broad to resolve chemical shift differences, the observed signal may also originate from the epoxy used to stabilize the NMR coil. Furthermore, the NMR linewidth is also broadened by temporal field drift within the 1-min NMR acquisition time. A separate drift analysis based on stabilized field periods at 1000 A indicated a temporal drift rate of ~350 ppm/hour (fig. S7), suggesting that spatial variation is the dominant contributor to the measured linewidth under static conditions. Hence, the 270-ppm FWHM serves as an upper boundary on the field nonuniformity over the effective sample volume.

Methods such as filamented HTS tape (*60*, *61*), current sweep cycles (*46*), and coil temperature regulation (*28*) could be implemented to reduce screening currents and improve field homogeneity. A coefficient of 4.9 T/V was obtained for the Hall sensor assuming linear behavior through the origin point (Fig. 3D). Last, ^1^H T_1_ relaxation time was measured at 20 T to confirm that the signal originated from the ^1^H nuclear spin rather than from a confounding or spurious source (Fig. 3F).

NMR experiments were conducted during a series of subsequent tests, in which the magnet was recharged to certain currents. The NMR linewidth of 270 ppm corresponds to magnetic field precisions of 14.033 ± 0.002 and 19.968 ± 0.003 T at the respective calibration points. The strong linearity observed in both the NMR-based calibration and the quantum Hall effect analysis supports the reliability of the Hall sensor at fields exceeding 33 T. By this time, however, the coil had undergone multiple quenches from operation errors, preventing it from being recharged to high fields.

## DISCUSSION

Many high-field magnets are designed with a long and large bore to accommodate the measurement apparatus, such as an NMR probe. However, compact all-HTS magnets with a small and short bore provide simpler and cheaper alternative architecture. As we have shown in this work, bulky components can be housed outside the magnet while locating only essential elements, such as the sample and detection coil, within the high-field region. Moreover, in applications such as NMR and materials characterization, where sample volumes are often in the millimeter range or smaller, there is a substantial potential for magnets to reduce bore sizes. Moreover, quadrupolar nuclei NMR and quantum material characterization can tolerate relatively lower field homogeneity while benefiting from higher magnetic fields (*8*). Small-bore magnets may also facilitate integration with emerging micro-NMR (spectrometer-on-a-chip), where radiofrequency coil dimensions below 1 mm and sample volumes down to the picoliter scale have been demonstrated (*62*–*65*).

One concern associated with small-bore HTS magnets is the mechanical strain imposed on the REBCO tape. If the strain exceeds the critical threshold, then the superconducting layer can crack, leading to reduced current-carrying capacity (*38*–*40*). Pancake coils are usually wound with a radius larger than 6 to 8 mm to avoid damage to the REBCO tape, while here, an exceptionally small diameter of 3.5 mm was used. Although a detailed mechanical analysis was not conducted, the integrity of the REBCO tape at this extreme curvature can be attributed to two factors: its geometry and the bending direction. Studies have shown that thinner REBCO tapes can tolerate smaller bending radius, especially when the superconducting layer faces inward, where it experiences compressive rather than tensile strain (*38*–*40*). Besides, unlike narrow tapes which are produced by slitting wider ones and exposing uncoated edges, the 12-mm tape retains its original protective coating on the edge, making it less prone to cracking (*18*).

We observed similar tape deformation, as reported in (*28*), in coil construction with extensive windings using thin REBCO tape. Variations in tape thickness, which accumulate with increasing turns, can cause deformation and overstrain, likely leading to the coil resistance observed during the helium test of the double-pancake coil. In addition, the magnets can experience strong Lorentz forces due to the extensive coil winding and high screening currents generated in the REBCO tape. Although a control experiment is needed to confirm this, the successful operation of these mini magnets at 40 T indicates that soldering the entire coil enhances mechanical stability, enabling the coils to withstand these intense forces during operation in liquid helium. This soldering approach functions similarly to the previously reported edge bonding method (*66*), where bonding the conductor edge to a mechanically stronger spacer altered boundary conditions and strain distribution, resulting in reduced maximum strains.

We have demonstrated that magnetic fields of up to 42 T can be achieved using all-HTS magnets, highlighting their potential for compact and accessible high-field magnet technology. Although these high fields come with a reduced magnet bore diameter, the short bore offers a practical advantage by allowing nonessential components to be positioned outside the bore. In the future, we aim to improve field homogeneity, extend direct NMR measurements beyond 40 T, and further advance magnet technology toward even higher magnetic fields.

## MATERIALS AND METHODS

Hall sensors (HE214SH/2) were purchased from Asensor Technology (Sweden). This Hall sensor was chosen because of its small sensor size (2 mm by 2 mm by 0.6 mm) that can fit into the 3.1-mm bore. The cryogenic temperature sensor (LS-CX-1030-SD-HT) was purchased from Lake Shore Cryogenics. High current dc power supplies (TSD10-2000/380) were purchased from MAGNA-POWER. Sn42/Bi57/Ag1 solder paste was purchased from CHIPQUIK. The stepper motor (MLN17A10-M06060P15000N-C6A0-XXX) was purchased from Thomson Linear. The NMR experiment used a pulse length of 0.25 μs and a recycle delay of 1 s. All NMR spectra were signal averaged for 64 scans. The longitudinal relaxation delay (T_1_) was measured using a saturation recovery experiment.

## Supplementary Materials

### This PDF file includes:

Supplementary Text Figs. S1 to S9 Tables S1 to S3 References
