# Supplementary material for: 40 Tesla miniature magnets
Source: Sci Adv. 2026 Mar 11;12(11):eadz5826. doi: 10.1126/sciadv.adz5826 (PMC12978251; doi:10.1126/sciadv.adz5826)
Supplement: Supplementary file 1 — Supplementary Text Figs. S1 to S9 Tables S1 to S3 References [file sciadv.adz5826_sm.pdf]

Science Advances
AAAS

# Supplementary Materials for

## 40 Tesla miniature magnets

Chukun Gao *et al.*

Corresponding author: Alexander B. Barnes, abarnes@ethz.ch

*Sci. Adv.* **12**, eadz5826 (2026)
DOI: 10.1126/sciadv.adz5826

**This PDF file includes:**

Supplementary Text
Figs. S1 to S9
Tables S1 to S3
References

**Supplementary Text**

Two smaller seamless double pancake coils

Two other smaller seamless double pancake coils (DP) manufactured using 20 m and 60 m long HTS tape, individually, were manufactured and tested prior to the 40 T double pancake coil described in the main text. Table S1 lists the key parameters for all three DP coils. DP 1 was the first trial of the seamless winding method and was wound onto a 5.0 mm mandrel. DP 2 and DP 3 both used 3.5 mm mandrel.

**Table S1. Key parameters for double pancake (DP) coils**

| coil | mandrel o.d. (mm) | coil o.d. (mm) | # of turns | HTS length (m) | R_coil (nΩ) | voltage decay time constant | max field & current | current density ($Amm^{-2}$) |
|---|---|---|---|---|---|---|---|---|
| DP 1 | 5.0 | 24.2/24.4 | 195/191 | 9.2/9.3 | 103/78 at 950 A | 5.2 min at 950 A | 23.9 T at 1656 A | 3000 |
| DP 2 | 3.5 | 42.2/42.1 | 415/420 | 30.5/30.8 | 105/253 at 800 A | 12.5 min at 800 A | 30.2 T at 1246 A | 2305 |
| DP 3 | 3.5 | 63.5/63.0 | 649/643 | 69.0/68.1 | 410/172 at 800 A | 1.92 h at 800 A | 38.3 T at 1248 A | 2278 |

Fig. S1 shows photographs and helium test results for DP 1. During the helium test, a charging rate of 0.01–0.1 A/s was employed. Nearly zero voltage was detected across the seamless connection, indicating no damage to the transitional turns around the 5 mm diameter mandrel. The resistances of the top and bottom coils were measured to be 103 nΩ and 78 nΩ, respectively, based on stabilized voltage values at 1000 A. At 1156 A, charging was paused due to an increase in the top coil voltage (30 seconds before the first quench). However, a larger voltage rise occurred 10 seconds before the first quench, and the magnet quenched at a magnetic field of 16.7 T.

In a second test conducted immediately after the first, DP 1 achieved a magnetic field of 23.9 T at 1656 A, corresponding to an overall current density of 3000 $A/mm^2$. Unlike the first quench, the second quench originated in the bottom coil, which showed a voltage increase 7 seconds before the quench.

Compared to the double pancake coil wound with same length of HTS tape in our previous work (*33*), DP 1 achieved a 58% higher current density (3000 $Amm^{-2}$ vs. 1900 $Amm^{-2}$) and a 50% stronger magnetic field (24 T vs. 16 T). This enhancement arises not only from the 20% increase in conductor width (12 mm vs. 10 mm), but more importantly from the seamless connection achieved in a smaller bore (3.5 mm vs. 8 mm). The elimination of a resistive joint within the bore suppresses heat generation and preserves the intrinsic current-carrying capacity of the REBCO tape.

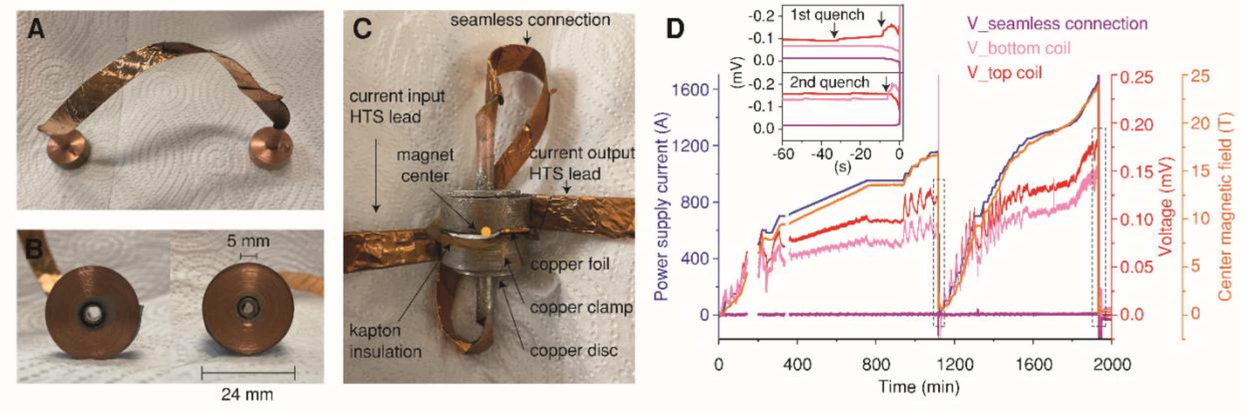


**Fig. S1. Fabrication and test result of DP 1 in liquid helium.** (**A**, **B**) Top and bottom views of DP 1 after winding. (**C**) Photograph of the soldered DP 1. (**D**) Test result of DP 1 in liquid helium.

Figure S2 presents photographs and test result of DP 2. During testing, a charging rate of 0.02–0.03 A/s was applied. Same as in DP 1, almost no voltage was detected across the seamless connection, confirming the absence of HTS tape damage at a bending diameter of 3.5 mm. The resistances of the top and bottom coils were measured to be 105 nΩ and 253 nΩ, respectively, based on stabilized voltage values at 800 A. Although the magnet was charged at 0.02 A/s near the quench, the significant inductive voltage drop observed at 1150 A suggested that this rate was still too high. The coil ultimately quenched when 1246 A, with a peak magnetic field of 31.2 T. In contrast to DP 1, no voltage increase was observed before the quench, suggesting that any such change may have occurred faster than the 10 Hz sampling rate of the measurement system.

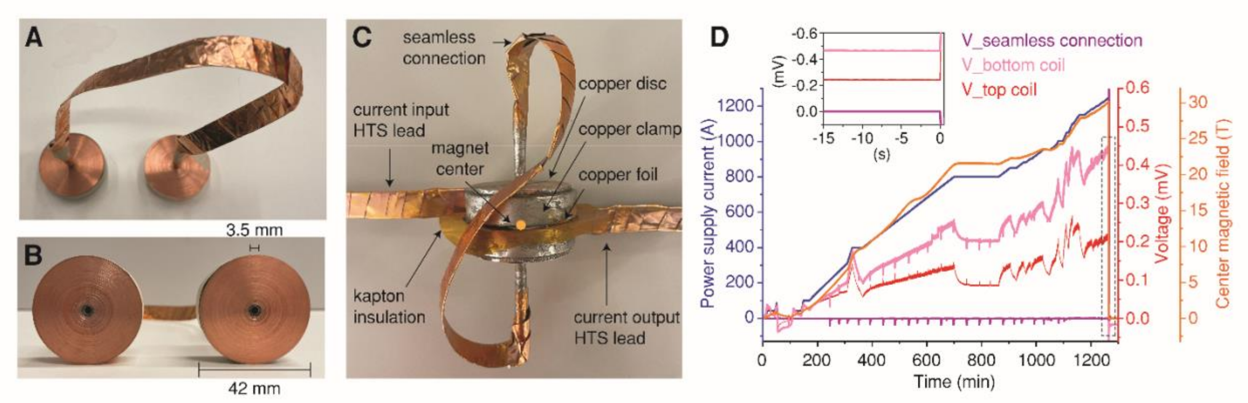


**Fig. S2. Fabrication and test result of DP 2 in liquid helium.** (**A**, **B**) Top and bottom views of DP 2 after winding. (**C**) Photograph of the soldered DP 2. (**D**) Test result of DP 2 in liquid helium. During the initial phase of the test (50–100 minutes), the magnet was rapidly discharged due to technical issues. Periodic voltage spikes observed in the data were caused by temperature fluctuations affecting insufficiently shielded measurement wires during automatic helium filling.

Figure S3 illustrates the Hall sensor measured magnetic field as a function of power supply current for all three DP coils. The dashed lines represent the simulated central magnetic field, Using a simple model assuming a uniform current distribution over the entire coil. The

discrepancy between the simulated and measured fields reflects the influence from bypass currents and the screening current induced field, which are more pronounced in larger coils with smaller bores.

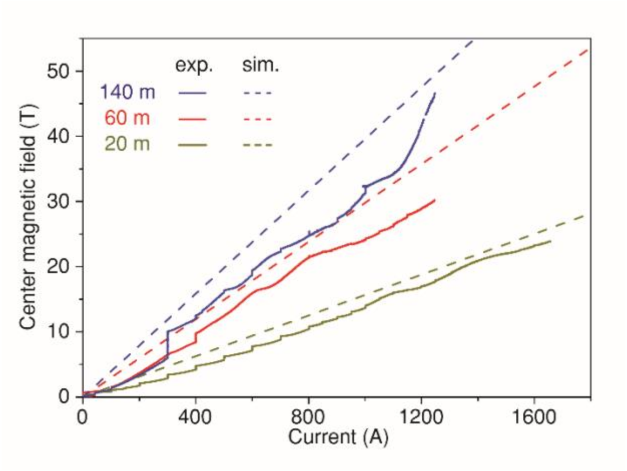


**Fig. S3. Experimental (solid line) and simulated (dash line) magnetic fields as a function of power supply current for all three seamless double pancake coils.**

---

Magnetic field fitting for the double pancake coil

A numerical model was developed to analyze the distribution of screening current based on the magnetic field profiles measured along the coil axis. The fitting was performed using the Phaedra code, which was originally developed for gyrotron design but also contains a magnetic field computation module. In previous work, the fitting model assumed a uniform operational current and two counterflowing screening currents evenly distributed over half the tape width (*33*). In this study, the model was refined to include more than two screening currents distributed across the tape width, with their sum constrained to zero. The coil was divided into evenly spaced grids along its axis, allowing for a more detailed current distribution analysis (fig. S4). The fitting yielded good agreement with the experimental result when using grid numbers of four or more (fig. S4A), with similar simulated currents (fig. S4B). While increasing the grid number allows for greater current variation across the tape, it significantly increases computation time. For instance, with a grid number of 20, the optimal result could not be obtained within 8 hours of simulation. To balance computation time with the ability to capture current variations, a grid number of 6 was selected for the simulation model.

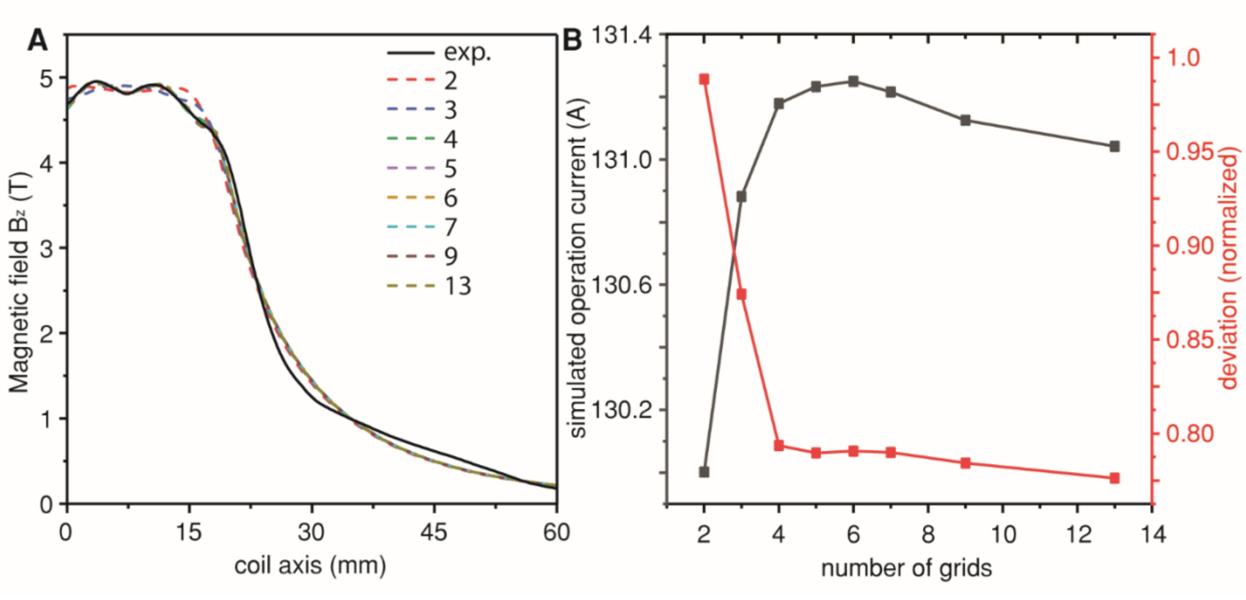


**Fig. S4. Optimization of fitting model using Phaedra code.** (**A**) Magnetic field profile fitting for measurements taken at 174.7 A. The dashed lines show the fitted results using different grid numbers. (**B**) Simulated operation current and deviation between fitting and measurement plotted as a function of grid number.

The Phaedra code employs a minimization error technique to determine the currents that yield an optimal fit. Figure S5A shows the fitting results for the magnetic field profiles measured at power supply currents ranging from 174.7 A to 805.5 A. The profiles were measured during charging with a rate between 0.005 A/s to 0.05 A/s. Only the field profile in region of 0 mm to 17 mm along the coil axis, where the motor moved smoothly, was used for fitting. For better visualization, the field profile at 447.2 A from fig. S5A is highlighted and displayed separately in fig. S5B. The observed discrepancies in regions farther from the coil center may stem from radial

current variations or tilting of the Hall sensor outside the magnet bore, factors that were not accounted for in the fitting process.

The calculated current distribution across the double pancake coil, corresponding to the fiting shown in fig. S5B, reveals a high concentration of screening current near the coil's outer edge (fig. S5C). The fitted operation current closely follows the trend of experimental center magnetic field (fig. S5D). The slight shift in the fitting at power supply current of 0.4 kA corresponds to a reduction in the ramping rate from 0.05 A/s to 0.005 A/s. Faster charging lead to a less stable current distribution, which accounts for the fitted operation current being lower than the power supply current.

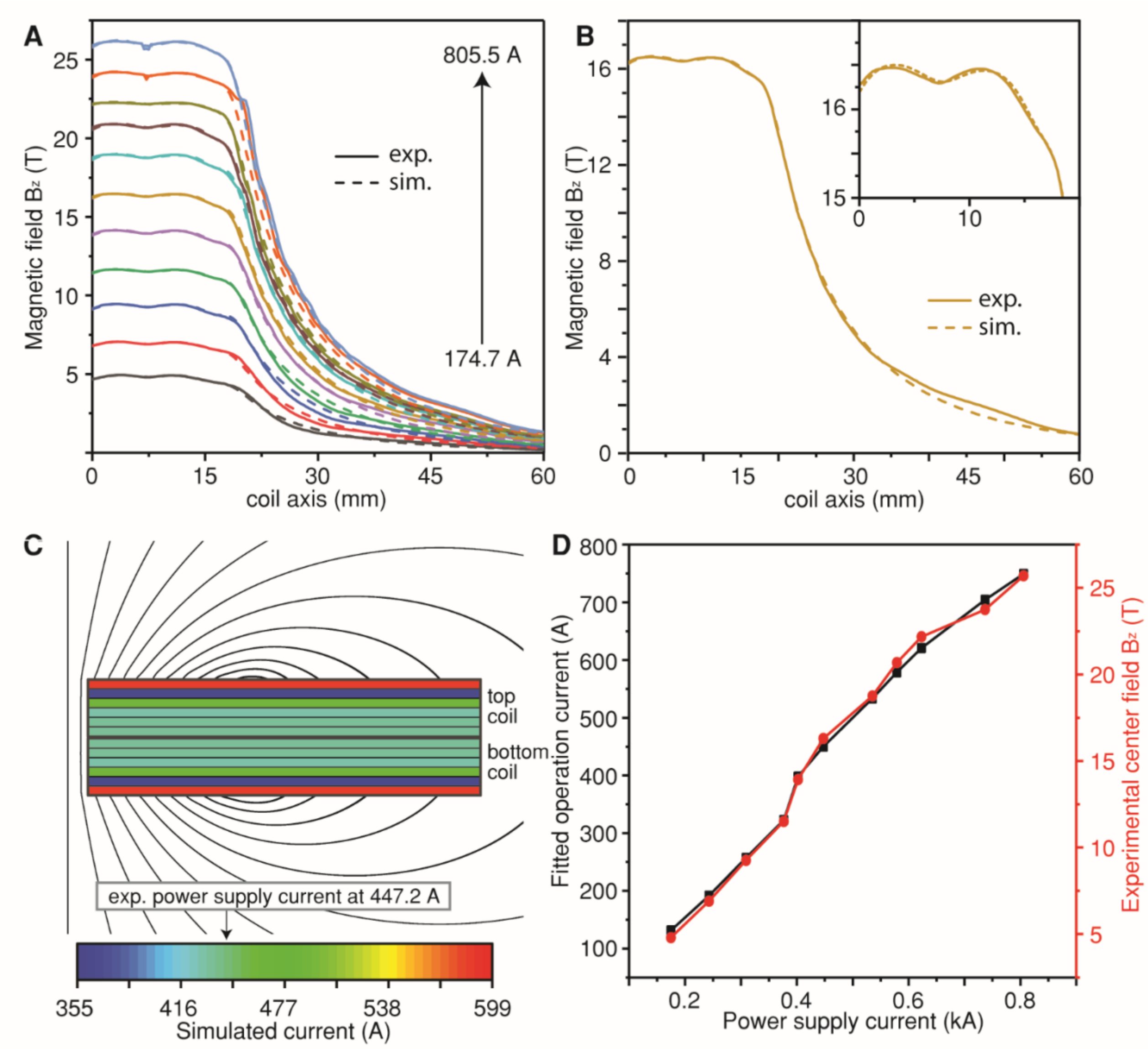


**Fig. S5. Fitting of magnetic field profiles measured by Hall sensor using Phaedra code.** (**A**) Comparison of fitted (dashed lines) and experimental (solid lines) magnetic field profiles for power supply currents ranging from 174.7 A to 805.5 A. (**B**) Expansion view of the field profile in (A) at power supply current of 447.2 A. (**C**) Fitted screening current distribution at 447.2 A,

shown in the section view of the double pancake coil (not to scale). (**D**) Fitted operation current and experimental center field as functions of power supply current.

Extended details for the fabrication and helium experiment of the double and quad pancake coils

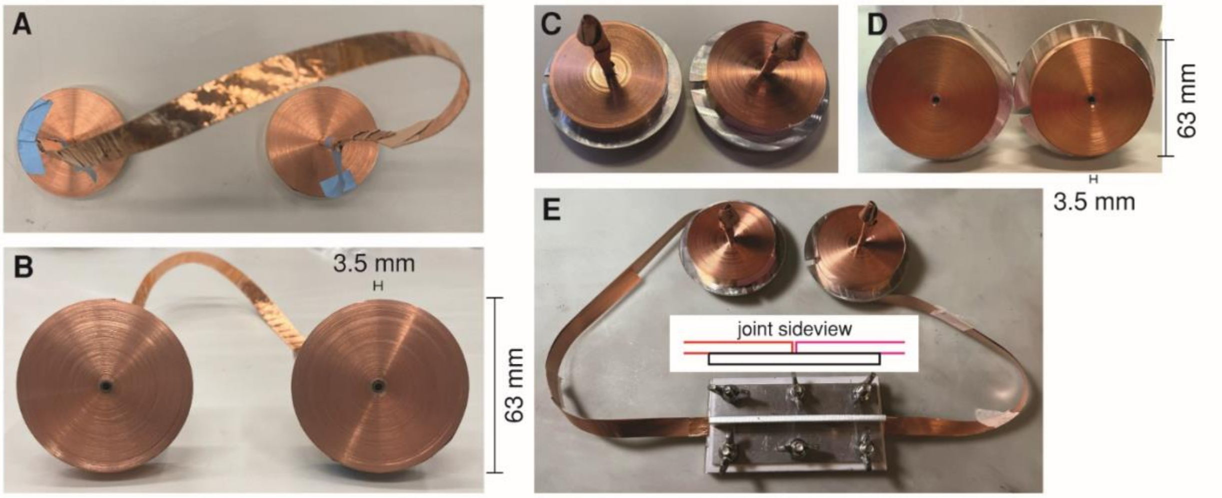


**Fig. S6. Photos of the double and quad pancake coil after winding.** (**A, B**) Top and bottom views of the two pancake coils used to assemble the seamless double pancake coil. (**C, D**) Top and bottom views of the two double pancake coils used to assemble the quad pancake coil. (**E**) The soldering process for the joint connecting the two seamless double pancake coils. Red indicates the original tape from the two coils, while black represents the additional tape section used to join them.

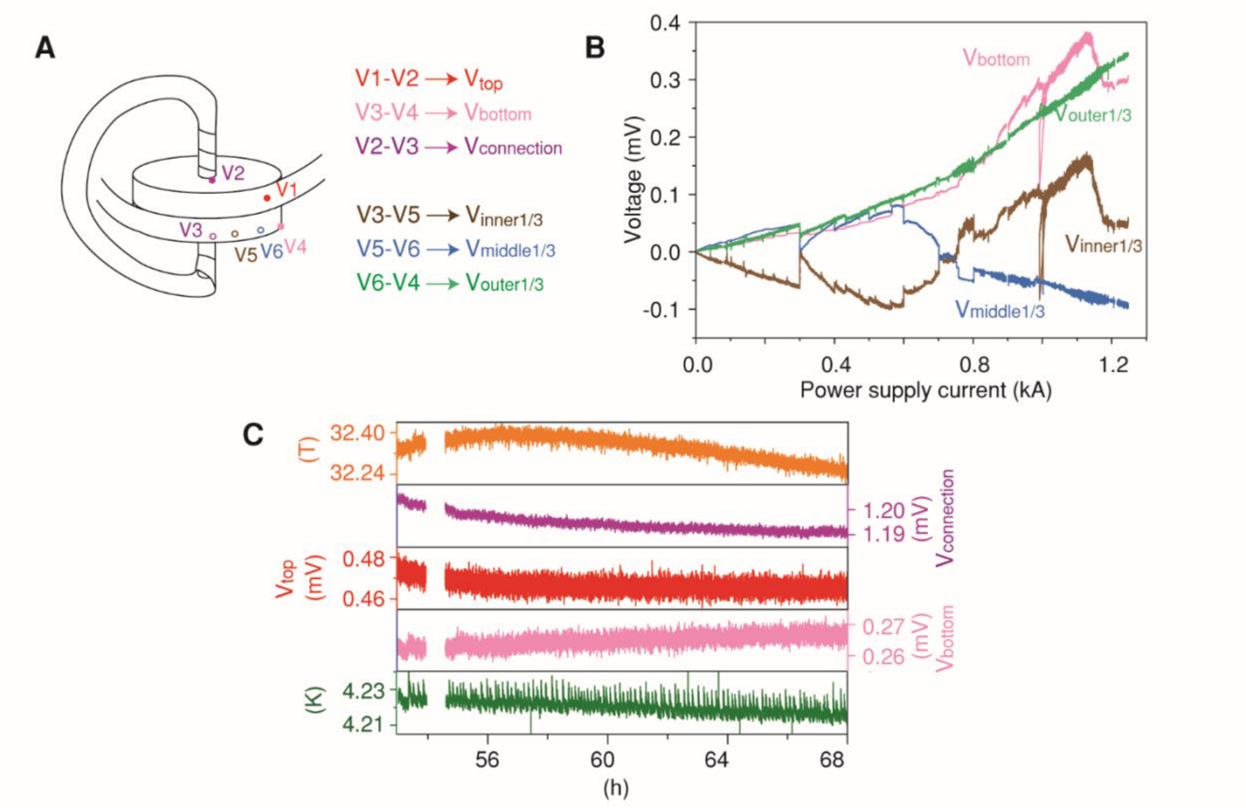


**Fig. S7. Helium test results of the double pancake coil.** (**A**) Arrangement of voltage taps on the double pancake coil. (**B**) Voltage of the bottom coil ($V_{bottom}$), along with partial voltages measured across the inner third ($V_{inner1/3}$), middle third ($V_{middle1/3}$), and outer third ($V_{outer1/3}$) of the coil, plotted as a function of the power supply current. The drop and recovery at 1 kA for $V_{bottom}$ and $V_{inner1/3}$ at 1 kA were caused by the discharge and subsequent recharge in response to a voltage spike (see expansion view in Fig. 2A). (**C**) Drift of voltages, temperature and magnetic field at static current of 1 kA over 14 hours after 5 hours of stabilization time. As expected, the coil temperature and voltages of the top coil and jointless connection gradually decreased, consistent with a greater proportion of the current transitioning into the superconductor due to coil inductance. Typically, at constant current, the magnetic field increases over time due to the relaxation of screening currents (*56*). However, in this case, the observed drift toward a lower field may result from minor current fluctuations within the deviation range of the power supply.

**Table S2. Voltage and resistance for the double pancake coil**

| current (A) | V(t) (h) | $R_{coil}$ (nΩ) | $R_{connection}$ (nΩ) |
|---|---|---|---|
| 300 | 5.26 | 274/77 | 834 |
| 800 | 1.92 | 410/172 | 1157 |
| 1000 | 1.79 | 462/262 | 1189 |

The voltage decay time constant V(t) at 300 A was 5.26 hours, more than twice as long as that at 800 A. This suggests a change in the contact resistance between the coil windings, resulting in a longer charging delay at lower currents (*43,44*). The overall coil resistance, calculated using the stabilized coil voltage divided by the power supply current, was higher at higher currents, could be due to microcracks caused to the REBCO tape under strong Lorenta forces and strains. The seamless connection showed a resistance of 1.2 μΩ at 1 kA, which resulted from an accident at the beginning of the coil winding. The tape was bent at 3.5 mm with REBCO layer facing outward**,** causing microcracks as the tensile strain exceeded the material limit (*41*, *42*). In other coils with similar winding diameters, the jointless connection showed nearly zero resistance up to 1600 A (fig. S1 and S2), indicating that the critical bending diameter is less than 3.5 mm when the HTS layer faces inward (*40-42*).

The double-pancake coil underwent 14 charging and thermal cycles in liquid helium. The initial coil resistances, 0.41 μΩ (top) and 0.17 μΩ (bottom), calculated from the stabilized voltage divided by current, increased gradually with each cycle, reaching 64 μΩ and 2.1 μΩ, respectively, after the 14th test. Although a detailed post-mortem examination to localize specific defects was not performed, the progressive increase in resistance suggests cumulative degradation over the entire coil in repeated charging cycles.

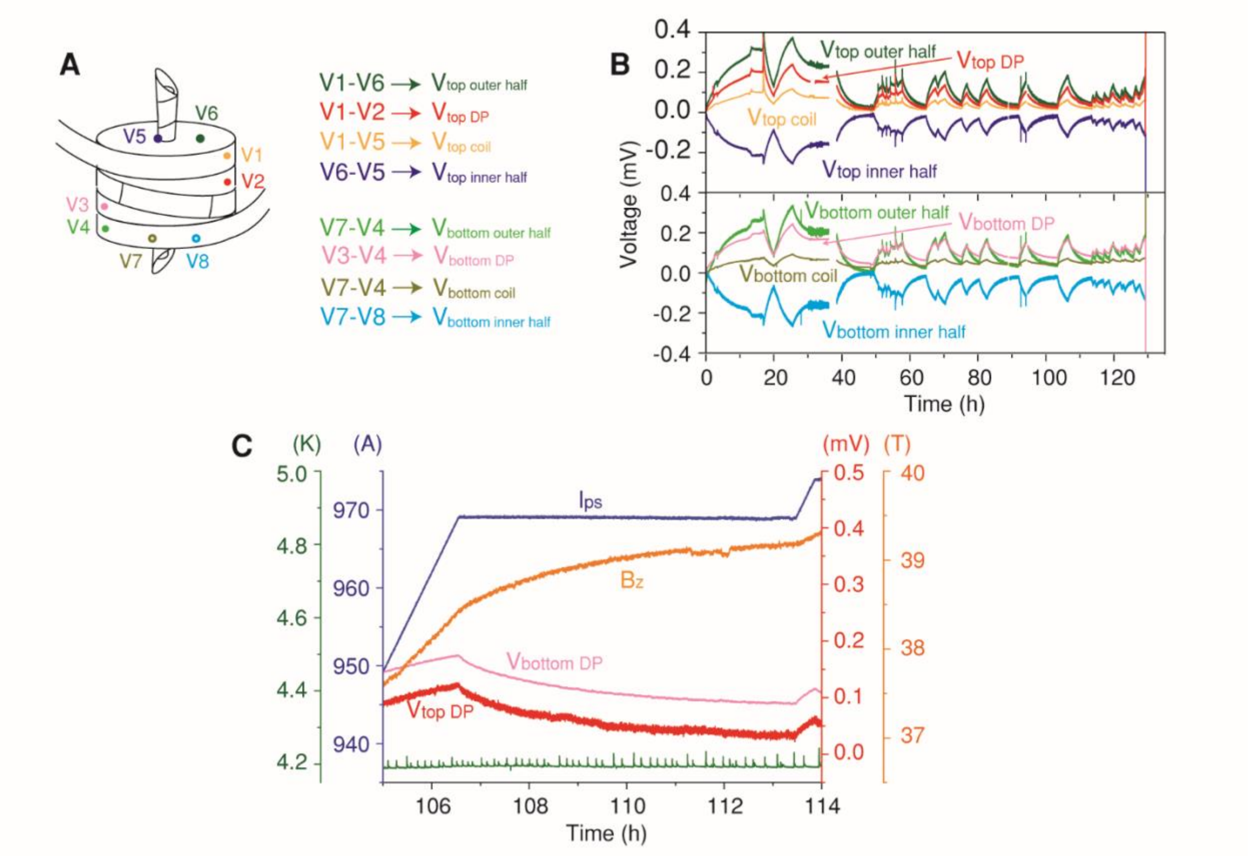


**Fig. S8. Helium test results for the quad pancake coil.** (**A**) Arrangement of voltage taps on the double pancake coil. (**B**) Partial voltages over the outermost (1st and 4th) pancake coils were measured by the voltage taps (V6 and V8) soldered at the middle turns. The inner halves of each pancake coil exhibited negative voltages, while the outer halves showed slightly larger positive voltages, resulting in a small net positive voltage measured across each pancake coil. The voltage across one double pancake unit was almost twice that of a single pancake coil, indicating similar voltage behavior across individual pancake coils and minimal voltage across the connections between them. (**C**) Magnetic field and voltage stabilization at 970 A.

Quantum Hall effect analysis

Consistent with previous work (*33*), features of quantum Hall effect were observed during the helium test, particularly when the Hall sensor was operated at a lower supply current, corresponding to a reduced electronic temperature. The quantum Hall effect emerges under conditions of low temperature and strong magnetic fields, where the Hall resistance $R_{xy}$ exhibits discrete, quantized plateaus given by:

$$R_{xy} = \frac{V_{Hall}}{I} = \frac{h}{e^2 \nu} = \frac{R_k}{\nu} \qquad \text{(S1)}$$

where $V_{Hall}$ is the Hall voltage, $I$ is the supply current to the Hall sensor, $h$ is the Plank constant, $e$ is the electron charge, and $R_K = h/e^2$ is the von Klitzing constant. The filling factor $\nu$ can take either integer or fractional values.

Hall sensor 1, operated with a constant 2 mA supply current through the entire magnetic field range, was positioned at the magnet center. Whereas sensor 2, operated with 0.2 mA and thus at consequently lower electronic temperature to better demonstrate the quantum Hall effect, was placed ~3 mm away. Figure S9 shows the Hall resistance $R_{xy}$ as a function of power supply current during the helium experiment. Sensor 2, running at the lower current, exhibited more pronounced quantized plateaus than sensor 1, likely due to reduced resistive heating and the resulting lower electronic temperature (*33,49*). Assuming that the Hall plateaus are symmetric with respect to the extrapolation of the classical low-field Hall effect, the magnetic field can be extracted from the midpoint of each plateau when assuming a carrier density in the sensor that was derived from the low field Hall effect (*70*).

Distinct plateaus at filling factor $\nu = 8$, 12, 16, and 28 were observed in the measurements from Hall sensor 2 (fig. S9 B). The observed degeneracy of 4 could be attributed to a two-layer system, incorporating both layer and spin degeneracy. However, since the Hall sensor manufacturer does not disclose details of the layer structure, the origin of the Landau level degeneracy cannot be definitively determined. In contrast, sensor 1, operated at a higher current of 2 mA, did not show clear plateaus, consistent with the suppression of quantum effects at elevated temperatures due to resistive heating (fig. S9 A).

During the charging, the supply current of sensor 2 was increased from 0.2 mA to 2 mA to raise its electronic temperature. Concurrently, although the supply current remained constant, sensor 1 began to show deviations in $R_{xy}$ from the expected linear trend. We attribute these changes to slow thermal equilibration between the sensors and their environment. Improved thermal isolation, such as increasing the distance between the sensors and enhancing encapsulation, could reduce temperature cross-influence and avoid quantum Hall effect in the future experiments.

Assuming a linear field–current relationship above 300 A, the midpoint of each current plateau corresponds to the center of the associated magnetic field plateau, where the classical and quantum Hall regimes intersect. A linear fit to these midpoints yields same slope for both sensors. Extrapolating this fit to the maximum power supply current gives an estimated peak magnetic field of 38.2 T, consistent with the 38.3 T obtained from the linear fit presented in the main manuscript.

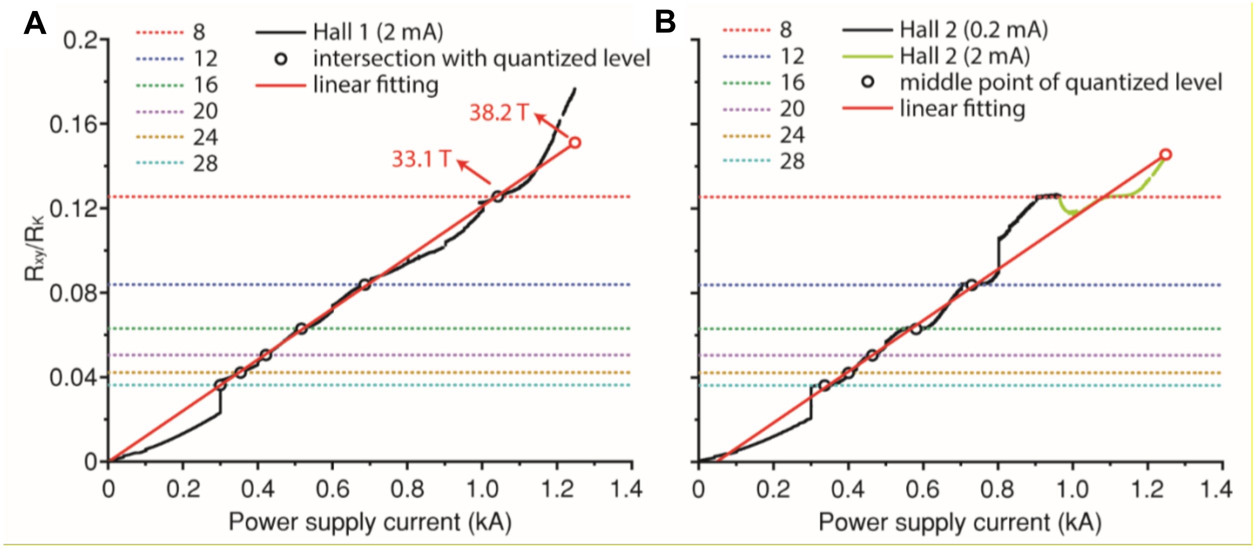


**Fig. S9. Quantum Hall effect analysis.** (**A**) Transverse resistance $R_{xy}$ measured by Hall sensor 1 with a supply current of 2 mA. (**B**) $R_{xy}$ measured by Hall sensor 2 with a supply current of 0.2 mA (black), and after switching to 2 mA near 1 kA (green). Dashed lines show quantized resistance based on the von Klitzing constant. The solid red line represents a linear fit to the midpoint values of the observed plateaus, or to their intersections where the plateaus are less distinct.

Representative comparison of high-field magnet parameters

Table S3 presents a comparison of selected high-field superconducting magnets, highlighting differences in field strength, bore size, conductor length, and engineering current density. In general, high-field magnets are achieved either by employing a strong background field (e.g., from a resistive or LTS magnet) or by utilizing long lengths of HTS conductor in large-scale windings. As the table shows, small-bore HTS magnets can achieve comparable or even higher field strengths with significantly shorter conductor lengths, enabling reduced fabrication cost and more affordable prototyping.

However, we recognize that miniature magnets have inherent limitations in practical applicability. Wide-bore, high-field magnets face unique and demanding engineering challenges, such as mechanical stress management, field homogeneity, and thermal stability, that must be addressed for applications such as NMR spectroscopy and fusion. Therefore, while direct comparisons between miniature and large-scale systems are constrained, compact HTS magnets still provide valuable insight into coil performance limits and offer a scalable platform for developing next-generation high-field magnet technologies.

**Table S3. Comparison of selected high-field superconducting magnets**

| Magnet | Type | Total field (T) | Background field (T) | Bore size (mm) | HTS length (m) | Current density in HTS (Amm$^{-2}$) |
|---|---|---|---|---|---|---|
| NHMFL 45.5 T hybrid (*16*) | HTS + resistive | 45.5 | 31.1 | 14 | 68 | 1,260 |
| ETH 42 T all-HTS (this work) | All HTS | 42.3 | 0 | 3.5 | 260 | 1,880 |
| ETH 38 T all-HTS (this work) | All HTS | 38.3 | 0 | 3.5 | 137 | 2,257 |
| China 32.35 T all-superconducting (*17*) | HTS + LTS | 32.35 | 15 | 43 | 2,133 | 150-330 |
| NHMFL 32 T T all-superconducting (*18*) | HTS + LTS | 32 | 15 | 40 | 3,233 | 170-190 |
| SuNAM 26 T all-HTS (*19*) | All HTS | 26 | 0 | 35 | 4,842 | 220-400 |
| MIT 25 T all-HTS (*20*) | All HTS | 25 | 0 | 12.5 | 750 | 680 |

**Note:** Background field refers to the magnetic field provided by an external source, separate from the HTS magnet. HTS lengths shown in table are converted to tape width of 12 mm.

## REFERENCES


1. J. R. Lewandowski, M. E. Halse, M. Blackledge, L. Emsley, Direct observation of hierarchical protein dynamics. *Science* **348**, 574–578 (2015).

2. J. Jeong, M. Kim, J. Seo, H. Lu, P. Ahlawat, A. Mishra, Y. Yang, M. A. Hope, F. T. Eickemeyer, M. Kim, Y. J. Yoon, I. W. Choi, B. P. Darwich, S. J. Choi, Y. Jo, J. H. Lee, B. Walker, S. M. Zakeeruddin, L. Emsley, U. Rothlisberger, A. Hagfeldt, D. S. Kim, M. Grätzel, J. Y. Kim, Pseudo-halide anion engineering for α-$FAPbI_3$ perovskite solar cells. *Nature* **592**, 381–385 (2021).

3. H. Han, S. J. Park, C. Sung, J. Kang, Y. H. Lee, J. Chung, T. S. Hahm, B. Kim, J. K. Park, J. G. Bak, M. S. Cha, G. J. Choi, M. J. Choi, J. Gwak, S. H. Hahn, J. Jang, K. C. Lee, J. H. Kim, S. K. Kim, W. C. Kim, J. Ko, W. H. Ko, C. Y. Lee, J. H. Lee, J. H. Lee, J. K. Lee, J. P. Lee, K. D. Lee, Y. S. Park, J. Seo, S. M. Yang, S. W. Yoon, Y. S. Na, A sustained high-temperature fusion plasma regime facilitated by fast ions. *Nature* **609**, 269–275 (2022).

4. J. Ongena, R. Koch, R. Wolf, H. Zohm, Magnetic-confinement fusion. *Nat. Phys.* **12**, 398–410 (2016).

5. P. F. Smith, D. B. Thomas, Superconducting magnets in high energy physics. *Nature* **216**, 964–969 (1967).

6. M. Derrick, Superconducting magnets in high-energy physics: Large-scale magnets that dissipate no electrical power are under construction for high-energy physics research. *Science* **158**, 325–331 (1967).

7. G. Diankov, C.-T. Liang, F. Amet, P. Gallagher, M. Lee, A. J. Bestwick, K. Tharratt, W. Coniglio, J. Jaroszynski, K. Watanabe, T. Taniguchi, D. Goldhaber-Gordon, Robust fractional quantum Hall effect in the $N$=2 Landau level in bilayer graphene. *Nat. Commun.* **7**, 13908 (2016).

8. Z. Gan, P. Gor'Kov, T. A. Cross, A. Samoson, D. Massiot, Seeking higher resolution and sensitivity for NMR of quadrupolar nuclei at ultrahigh magnetic fields. *J. Am. Chem. Soc.* **124**, 5634–5635 (2002).

9. Z. J. Berkson, S. Björgvinsdóttir, A. Yakimov, D. Gioffrè, M. D. Korzyński, A. B. Barnes, C. Copéret, Solid-state NMR spectra of protons and quadrupolar nuclei at 28.2 T: Resolving signatures of surface sites with fast magic angle spinning. *JACS Au* **2**, 2460–2465 (2022).

10. H. Maeda, Y. Yanagisawa, Recent developments in high-temperature superconducting magnet technology (Review). *IEEE Trans. Appl. Supercond.* **24**, 1–12 (2014).

11. T. Kiyoshi, S. Choi, S. Matsumoto, K. Zaitsu, T. Hase, T. Miyazaki, A. Otsuka, M. Yoshikawa, M. Hamada, M. Hosono, Y. Yanagisawa, H. Nakagome, M. Takahashi, T. Yamazaki, H. Maeda, HTS-NMR: Present status and future plan. *IEEE Trans. Appl. Supercond.* **20**, 714–717 (2010).

12. S. Hahn, D. K. Park, J. Bascuñán, Y. Iwasa, HTS pancake coils without turn-to-turn insulation. *IEEE Trans. Appl. Supercond.* **21**, 1592–1595 (2011).

13. S. Choi, H. C. Jo, Y. Hwang, S. Hahn, T. Ko, A study on the no insulation winding method of the HTS coil. *IEEE Trans. Appl. Supercond.* **22**, 4904004 (2012).

14. S. Hahn, D. K. Park, J. Voccio, J. Bascunan, Y. Iwasa, No-insulation (NI) HTS inserts for >1 GHz LTS/HTS NMR magnets. *IEEE Trans. Appl. Supercond.* **22**, 4302405 (2012).

15. Y. Li, D. Hu, J. Zhang, W. Wu, Z. Li, K. Ryu, Z. Hong, Z. Jin, Feasibility study of the impregnation of a no-insulation HTS coil using solder. *IEEE Trans. Appl. Supercond.* **28**, 1–5 (2018).

16. Y. Q. Li, M. Yin, J. W. Zhang, Z. Y. Li, Z. Hong, Z. Jin, Study on reducing the charge delay of the no-insulation HTS coil after solder impregnation. *Phys. C Supercond. Appl.* **552**, 42–47 (2018).

17. H.-J. Shin, K. L. Kim, Y. H. Choi, O. J. Kwon, S. Hahn, Y. Iwasa, H. G. Lee, Effects of impregnating materials on thermal and electrical stabilities of the hts racetrack pancake coils without turn-to-turn insulation. *IEEE Trans. Appl. Supercond.* **23**, 7700404 (2013).

18. S. Hahn, K. Kim, K. Kim, X. Hu, T. Painter, I. Dixon, S. Kim, K. R. Bhattarai, S. Noguchi, J. Jaroszynski, D. C. Larbalestier, 45.5-Tesla direct-current magnetic field generated with a high-temperature superconducting magnet. *Nature* **570**, 496–499 (2019).

19. M. D. Bird, I. R. Dixon, J. Toth, Large, high-field magnet projects at the NHMFL. *IEEE Trans. Appl. Supercond.* **25**, 1–6 (2015).

20. Z. Gan, I. Hung, X. Wang, J. Paulino, G. Wu, I. M. Litvak, P. L. Gor'kov, W. W. Brey, P. Lendi, J. L. Schiano, M. D. Bird, I. R. Dixon, J. Toth, G. S. Boebinger, T. A. Cross, NMR spectroscopy up to 35.2 T using a series-connected hybrid magnet. *J. Magn. Reson.* **284**, 125–136 (2017).

21. J. Toth, S. T. Bole, Design, construction, and first testing of a 41.5 T all-resistive magnet at the NHMFL in tallahassee. *IEEE Trans. Appl. Supercond.* **28**, 1–4 (2018).

22. J. R. Miller, "The NHMFL 45-T hybrid magnet system: Past, present, and future," in *IEEE Transactions on Applied Superconductivity* (IEEE, 2003), vol. 13, pp. 1385–1390.

23. J. Liu, Q. Wang, L. Qin, B. Zhou, K. Wang, Y. Wang, L. Wang, Z. Zhang, Y. Dai, H. Liu, X. Hu, H. Wang, C. Cui, D. Wang, H. Wang, J. Sun, W. Sun, L. Xiong, World record 32.35 tesla direct-current magnetic field generated with an all-superconducting magnet. *Supercond. Sci. Technol.* **33**, 03LT01 (2020).

24. P. C. Michael, D. Park, Y. H. Choi, J. Lee, Y. Li, J. Bascunan, S. Noguchi, S. Hahn, Y. Iwasa, Assembly and test of a 3-nested-coil 800-MHz REBCO insert (H800) for the MIT 1.3 GHz LTS/HTS NMR magnet. *IEEE Trans. Appl. Supercond.* **29**, 1–6 (2019).

25. Y. Suetomi, T. Yoshida, S. Takahashi, T. Takao, G. Nishijima, H. Kitaguchi, Y. Miyoshi, M. Hamada, K. Saito, R. Piao, Y. Takeda, H. Maeda, Y. Yanagisawa, Quench and self-protecting behaviour of an intra-layer no-insulation (LNI) REBCO coil at 31.4 T. *Supercond. Sci. Technol.* **34**, 064003 (2021).

26. A. H. Al-Tawhid, S. J. Poage, S. Salmani-Rezaie, A. Gonzalez, S. Chikara, D. A. Muller, D. P. Kumah, M. N. Gastiasoro, J. Lorenzana, K. Ahadi, Enhanced critical field of superconductivity at an oxide interface. *Nano Lett.* **23**, 6944–6950 (2023).

27. S. Yoon, J. Kim, K. Cheon, H. Lee, S. Hahn, S.-H. Moon, 26 T 35 mm all-$GdBa_2Cu_3O_{7-x}$ multi-width no-insulation superconducting magnet. *Supercond. Sci. Technol.* **29**, 04LT04 (2016).

28. D. Park, W. Lee, J. Bascuñán, H. M. Kim, Y. Iwasa, A cryogen-free 25-T REBCO magnet with the extreme-no-insulation winding technique. *IEEE Trans. Appl. Supercond.* **32**, 4602305 (2022).

29. Z. S. Hartwig, R. F. Vieira, D. Dunn, T. Golfinopoulos, B. LaBombard, C. J. Lammi, P. C. Michael, S. Agabian, D. Arsenault, R. Barnett, M. Barry, L. Bartoszek, W. K. Beck, D. Bellofatto, D. Brunner, W. Burke, J. Burrows, W. Byford, C. Cauley, S. Chamberlain, D. Chavarria, J. Cheng, J. Chicarello, V. Diep, E. Dombrowski, J. Doody, R. Doos, B. Eberlin, J. Estrada, V. Fry, M. Fulton, S. Garberg, R. Granetz, A. Greenberg, M. Greenwald, S. Heller, A. E. Hubbard, E. Ihloff, J. H. Irby, M. Iverson, P. Jardin, D. Korsun, S. Kuznetsov, S. Lane-Walsh, R. Landry, R. Lations, R. Leccacorvi, M. Levine, G. Mackay, K. Metcalfe, K. Moazeni, J. Mota, T. Mouratidis, R. Mumgaard, J. Muncks, R. A. Murray, D. Nash, B. Nottingham, C. O'Shea, A. T. Pfeiffer, S. Z. Pierson, C. Purdy, A. Radovinsky, D. K. Ravikumar, V. Reyes, N. Riva, R. Rosati, M. Rowell, E. E. Salazar, F. Santoro, A. Sattarov, W. Saunders, P. Schweiger, S. Schweiger, M. Shepard, S. Shiraiwa, M. Silveira, F. Snowman, B. N. Sorbom, P. Stahle, K. Stevens, J. Stillerman, D. Tammana, T. L. Toland, D. Tracey, R. Turcotte, K. Uppalapati, M. Vernacchia, C. Vidal, E. Voirin, A. Warner, A. Watterson, D. G. Whyte, S. Wilcox, M. Wolf, B. Wood, L. Zhou, A. Zhukovsky, The SPARC toroidal field model coil program. *IEEE Trans. Appl. Supercond.* **34**, 0600316 (2023).

30. Z. Y. Li, Z. C. Pan, H. G. Yang, Y. Y. Li, Y. J. Cao, L. Qiao, B. Gao, G. Huang, C. Zhang, K. P. Zhu, Y. S. Zhao, K. F. Chen, J. Q. Zhou, L. Yao, Q. Q. Wei, Y. X. Guo, Y. Y. Liu, Y. Huang, H. Qiao, W. J. Chen, Y. Q. Du, K. Zhang, X. Chen, A. H. Gong, G. Dong, Y. M. Ye, Z. Yang, 21.7-T large-scale high-temperature superconducting toroidal magnet for tokamak fusion application. *IEEE Trans. Appl. Supercond.* **35**, 1–6 (2025).

31. P. H. Chen, C. Gao, N. Alaniva, S. Björgvinsdóttir, I. G. Pagonakis, M. A. Urban, A. Däpp, R. Gunzenhauser, A. B. Barnes, Watch-sized 12 Tesla all-high-temperature-superconducting magnet. *J. Magn. Reson.* **357**, 107588 (2023).

32. C. Gao, P.-H. Chen, N. Alaniva, S. Björgvinsdóttir, I. Pagonakis, A. Däpp, M. Urban, R. Gunzenhauser, A. Barnes, 23 Tesla high temperature superconducting pocket *magnet. Supercond. Sci. Technol.* **37**, 065018 (2024).

33. C. Gao, P.-H. Chen, N. Alaniva, S. Björgvinsdóttir, I. Pagonakis, A. Däpp, M. Urban, R. Gunzenhauser, A. Barnes, Fabrication procedures and mechanical supports of no-insulation All-GdBCO double pancake magnets in liquid helium. *IEEE Trans. Appl. Supercond.* **34**, 1–9 (2024).

34. A. W. Zimmermann, S. M. Sharkh, "Design of a 1 MJ/100 kW high temperature superconducting magnet for energy storage," in *Energy Reports* (Elsevier Ltd., 2020), vol. 6, pp. 180–188.

35. R. Miyao, H. Igarashi, A. Ishiyama, S. Noguchi, Thermal and electromagnetic simulation of multistacked no-insulation REBCO pancake coils on normal-state transition by PEEC method. *IEEE Trans. Appl. Supercond.* **28**, 1–5 (2018).

36. M. Yoon, S. Lee, J. K. Lee, G. W. Hong, K. Choi, W. S. Kim, A concentrically arranged joint-less HTS coil system for persistent current mode operation. *IEEE Trans. Appl. Supercond.* **30**, 1–5 (2020).

37. J. Kang, T. K. Ko, E. A. Al-Ammar, K. Hur, Jointless pancake coil winding for minimizing electrical loss in HTS SMES for wind power. *IEEE Trans. Appl. Supercond.* **25**, 1–5 (2015).

38. S. C. Richter, D. Schoerling, S. S. I Schlachter, B. Ringsdorf, A. Drechsler, A. Bernhard, A.-S. Müller, *Bending Radius Limits of Different Coated REBCO Conductor Tapes—An Experimental Investigation with Regard to HTS Undulators* (2021).

39. Y. Shi, S. Dai, T. Ma, W. Liu, Effect of bending strain on the current-carrying performance of copper-laminated REBCO tape. *J. Supercond. Nov. Magn.* **35**, 647–655 (2022).

40. S. Yuan, T. Ma, S. Dai, M. Song, L. Li, Bending characteristics of stacked REBCO cable under different stacking modes. *Supercond. Sci. Technol.* **36**, 095007 (2023).

41. T. Sung Lee, Y. Jin Hwang, J. Lee, W. Seung Lee, J. Kim, S. Hyun Song, M. Cheol Ahn, T. Kuk Ko, The effects of co-wound Kapton, stainless steel and copper, in comparison with no insulation, on the time constant and stability of GdBCO pancake coils. *Supercond. Sci. Technol.* **27**, 065018 (2014).

42. S. B. Kim, T. Kaneko, H. Kajikawa, J. H. Joo, J.-M. Jo, Y.-J. Han, H.-S. Jeong, The transient stability of HTS coils with and without the insulation and with the insulation being replaced by brass tape. *IEEE Trans. Appl. Supercond.* **23**, 7100204 (2013).

43. K. L. Kim, S. Hahn, Y. Kim, D. G. Yang, J.-B. Song, J. Bascuñán, H. Lee, Y. Iwasa, Effect of winding tension on electrical behaviors of a no-insulation ReBCO pancake coil. *IEEE Trans. Appl. Supercond.* **24**, 1–5 (2014).

44. X. Wang, S. Hahn, Y. Kim, J. Bascuñán, J. Voccio, H. Lee, Y. Iwasa, Turn-to-turn contact characteristics for an equivalent circuit model of no-insulation ReBCO pancake coil. *Supercond. Sci. Technol.* **26**, 035012 (2013).

45. Z. Zhang, K. Wang, X. Wang, S. Chen, H. Suo, L. Ma, J. Liu, L. Wang, Q. Wang, Effect of the Cu stabilisation layer on the turn-to-turn contact resistance of a non-insulated REBCO winding. *Phys. C Supercond. Appl.* **590**, 1353949 (2021).

46. Y. Yanagisawa, Y. Kominato, H. Nakagome, T. Fukuda, T. Takematsu, T. Takao, M. Takahashi, H. Maeda, "Effect of coil current sweep cycle and temperature change cycle on the screening current-induced magnetic field for ybco-coated conductor coils," in *AIP Conference Proceedings* (Advances in Cryogenic Engineering, 2012), vol. 1434, pp. 1373–1380.

47. J. Matthews, M. E. Cage, Temperature dependence of the Hall and longitudinal resistances in a quantum Hall resistance standard. *J. Res. Natl. Inst. Stand. Technol.* **110**, 497–510 (2005).

48. D. Tong, Lectures on the Quantum Hall Effect. (2016).

49. K. R. Bhattarai, K. Kim, K. Kim, K. Radcliff, X. Hu, C. Im, T. Painter, I. Dixon, D. Larbalestier, S. Lee, S. Hahn, Understanding quench in no-insulation (NI) REBCO magnets through experiments and simulations. *Supercond. Sci. Technol.* **33**, 035002 (2020).

50. D. Park, J. Bascuñán, P. C. Michael, J. Lee, Y. H. Choi, Y. Li, S. Hahn, Y. Iwasa, MIT 1.3-GHz LTS/HTS NMR magnet: Post quench analysis and new 800-MHz insert design. *IEEE Trans. Appl. Supercond.* **29**, 1–4 (2019).

51. H. W. Weijers, W. D. Markiewicz, A. V. Gavrilin, A. J. Voran, Y. L. Viouchkov, S. R. Gundlach, P. D. Noyes, D. V. Abraimov, H. Bai, S. T. Hannahs, T. P. Murphy, Progress in the development and construction of a 32-T superconducting magnet. *IEEE Trans. Appl. Supercond.* **26**, 1–7 (2016).

52. H. Maeda, T. Yamazaki, Y. Nishiyama, M. Hamada, K. Hashi, T. Shimizu, H. Suematsu, Y. Yanagisawa, Development of super-high-field NMR operated beyond 1 GHz using high-temperature superconducting coils. *eMagRes* **5**, 1109–1120 (2016).

53. N. Amemiya, K. Akachi, Magnetic field generated by shielding current in high Tc superconducting coils for NMR magnets. *Supercond. Sci. Technol.* **21**, 095001 (2008).

54. Y. Yan, Y. Li, T. Qu, Screening current induced magnetic field and stress in ultra-high-field magnets using REBCO coated conductors. *Supercond. Sci. Technol.* **35**, 014003 (2022).

55. S. Tumanski, *Handbook of Magnetic Measurements* (Taylor & Francis Group, ed. 1, 2011; https://taylorfrancis.com/books/mono/10.1201/b10979/handbook-magnetic-measurements-slawomir-tumanski).

56. C. Germain, Bibliographical review of the methods of measuring magnetic fields. *Nucl. Instrum. Methods* **21**, 17–46 (1963).

57. J. Schaefer, K. Blvd, Multi-tuned single coil transmission line probe for nuclear magnetic resonance spectrometer (1997).

58. B. Gizatullin, C. Mattea, S. Stapf, Hyperpolarization by DNP and molecular dynamics: Eliminating the radical contribution in NMR relaxation studies. *J. Phys. Chem. B* **123**, 9963–9970 (2019).

59. S. Lange, A. H. Linden, Ü. Akbey, W. Trent Franks, N. M. Loening, B.-J. Van Rossum, H. Oschkinat, The effect of biradical concentration on the performance of DNP-MAS-NMR. *J. Magn. Reson.* **216**, 209–212 (2012).

60. E. Berrospe-Juarez, F. Trillaud, V. M. R. Zermeño, F. Grilli, "Screening current-induced field and field drift study in HTS coils using T-A homogenous model," in *Journal of Physics: Conference Series* (Institute of Physics Publishing, 2020), vol. 1559, 1–12.

61. Y. Yanagisawa, Y. Xu, X. Jin, H. Nakagome, H. Maeda, Reduction of screening current-induced magnetic field of REBCO coils by the use of multi-filamentary tapes. *IEEE Trans. Appl. Supercond.* **25**, 1–5 (2015).

62. D. L. Olson, T. L. Peck, A. G. Webb, R. L. Magin, J. V. Sweedler, High-resolution microcoil $^{1}$H-NMR for mass-limited, nanoliter-volume samples. *Science* **270**, 1967–1970 (1995).

63. N. Sahin Solmaz, R. Farsi, G. Boero, 200 GHz single chip microsystems for dynamic nuclear polarization enhanced NMR spectroscopy. *Nat. Commun.* **15**, 5485 (2024).

64. K. R. Minard, R. A. Wind, Picoliter $^{1}$H NMR spectroscopy. *J. Magn. Reson.* **154**, 336–343 (2002).

65. C. Massin, F. Vincent, A. Homsy, K. Ehrmann, G. Boero, P. A. Besse, A. Daridon, E. Verpoorte, N. F. De Rooij, R. S. Popovic, Planar microcoil-based microfluidic NMR probes. *J. Magn. Reson.* **164**, 242–255 (2003).

66. Y. Yan, P. Song, C. Xin, M. Guan, Y. Li, H. Liu, T. Qu, Screening-current-induced mechanical strains in REBCO insert coils. *Supercond. Sci. Technol.* **34**, 085012 (2021).
